# Genomic Analysis Confirms Population Structure and Identifies Inter-Lineage Hybrids in *Aegilops tauschii*

**DOI:** 10.3389/fpls.2019.00009

**Published:** 2019-01-25

**Authors:** Narinder Singh, Shuangye Wu, Vijay Tiwari, Sunish Sehgal, John Raupp, Duane Wilson, Mehraj Abbasov, Bikram Gill, Jesse Poland

**Affiliations:** ^1^Department of Plant Pathology, Wheat Genetics Resource Center, Kansas State University, Manhattan, KS, United States; ^2^Department of Plant Science & Landscape Architecture, University of Maryland, College Park, MD, United States; ^3^Department of Agronomy, Horticulture and Plant Science, South Dakota State University, Brookings, SD, United States; ^4^Genetic Resources Institute, Azerbaijan National Academy of Sciences, Baku, Azerbaijan

**Keywords:** *Aegilops tauschii*, genotyping-by-sequencing, inter-lineage hybrid, population structure, single nucleotide polymorphism, *Triticum aestivum*

## Abstract

*Aegilops tauschii*, the D-genome donor of bread wheat, *Triticum aestivum*, is a storehouse of genetic diversity, and an important resource for future wheat improvement. Genomic and population analysis of 549 *Ae. tauschii* and 103 wheat accessions was performed by using 13,135 high quality SNPs. Population structure, principal component, and cluster analysis confirmed the differentiation of *Ae. tauschii* into two lineages; lineage 1 (L1) and lineage 2 (L2), the latter being the wheat D-genome donor. Lineage L1 contributes only 2.7% of the total introgression from *Ae. tauschii* for a set of United States winter wheat lines, confirming the great amount of untapped genetic diversity in L1. Lineage L2 accessions had overall greater allelic diversity and wheat accessions had the least allelic diversity. Both lineages also showed intra-lineage differentiation with L1 being driven by longitudinal gradient and L2 differentiated by altitude. There has previously been little reported on natural hybridization between L1 and L2. We found nine putative inter-lineage hybrids in the population structure analysis, each containing numerous lineage-specific private alleles from both lineages. One hybrid was confirmed as a recombinant inbred between the two lineages, likely artificially post collection. Of the remaining eight putative hybrids, a group of seven from Georgia carry 713 SNPs with private alleles, which points to the possibility of a novel L1–L2 hybrid lineage. To facilitate the use of *Ae. tauschii* in wheat improvement, a MiniCore consisting of 29 L1 and 11 L2 accessions, has been developed based on genotypic, phenotypic and geographical data. MiniCore reduces the collection size by over 10-fold and captures 84% of the total allelic diversity in the whole collection.

## Introduction

World population is projected to reach 9.7 billion by 2050, increasing pressure on the food system and challenging food security (Fao et al., 2014). Wheat, among other major food crops, is currently at an estimated genetic gain of 1% per year. This must more than double to achieve the estimated 2.4% per year to meet the projected production levels needed to provide enough calories and protein to the billions around the world in the coming decades (Ray et al., 2013). However, limited genetic diversity present in the elite wheat cultivars pose a serious threat to this goal (Akhunov et al., 2010). To mitigate this genetic diversity problem, use of crop wild relatives and progenitors, such as goat grass (*Ae. tauschii* Coss.), presents a promising solution and the best resource.

*Aegilops tauschii* originated as the result of hybridization between diploid A and B genome progenitors (Marcussen et al., 2014), and became the diploid D-genome donor of bread wheat (*Triticum aestivum* L.). *Ae. tauschii* is native throughout the Caspian Sea region and into central Asia and China. Natural hybridization of tetraploid wheat and *Ae. tauschii* about 8,000–10,000 years ago (Renfrew, 1973; Bell, 1987) led to the formation of hexaploid wheat with *Ae. tauschii* contributing many genes that expanded the climatic adaption and improved bread making quality (Kihara, 1944; McFadden and Sears, 1946; Yamashita et al., 1957; Kerber and Tipples, 1969; Lagudah et al., 1991). However, during bread wheat evolution, only a handful of *Ae. tauschii* accessions from a small region hybridized with wheat leading to a narrow genetic base of the wheat D genome (Lagudah et al., 1991). Multiple studies have corroborated this, showing that the D-genome of wheat has the least genetic diversity as compared to its counterparts, A and B genomes (Kam-Morgan et al., 1989; Lubbers et al., 1991; Akhunov et al., 2010). However, much greater genetic diversity is present in this wild donor of the D-genome (Naghavi et al., 2009).

With a pressing need to develop better yielding wheat varieties to feed a growing population and adapt to a changing climate, *Ae. tauschii* is a valuable source of novel alleles for wheat improvement (Kihara, 1944; Lagudah et al., 1991). *Aegilops tauschii* harbors considerable genetic diversity for diseases and abiotic factors relative to the wheat D-genome, and is split into two subspecies known as *Ae. tauschii* ssp. *tauschii* (Lineage 1; L1) and ssp. *strangulata* (Lineage 2; L2). The L2 ssp. *strangulata* is known to be the D-genome donor (Jaaska, 1978; Nakai, 1979; Nishikawa et al., 1980; Jaaska, 1981). Ssp. *tauschii* is further split into three varieties- *typica*, *anathera*, and *meyeri*, whereas ssp. *strangulata* is monotypic. Phenotypic classification of these subspecies, especially to varieties, is challenging. Therefore phenotypic data often poorly correlate with genetic classification (Lubbers et al., 1991; Dvorak et al., 1998).

Genetic diversity present in *Ae. tauschii* has been utilized via synthetic hybridization of tetraploid wheat and wild *Ae. tauschii* (McFadden and Sears, 1945; Kihara and Lilienfeld, 1949), and introgressed to bread wheat through direct crossing (Gill and Raupp, 1987). However, considerable amounts of untapped genetic diversity remain present in this species. In this study, we characterized the full *Ae. tauschii* collection held at Wheat Genetics Resource Center at Kansas State University in Manhattan, KS, United States with the main objectives to genetically characterize the *Ae. tauschii* collection, study the population structure within *Ae. tauschii*, and develop a genetically diverse MiniCore set to facilitate the use of *Ae. tauschii* for wheat improvement. In conclusion, we present a strategy to utilize the genetic diversity from *Ae. tauschii* to broaden the genetic base of D-genome of hexaploid wheat.

## Materials and Methods

### Plant Material

**FIGURE 1:**
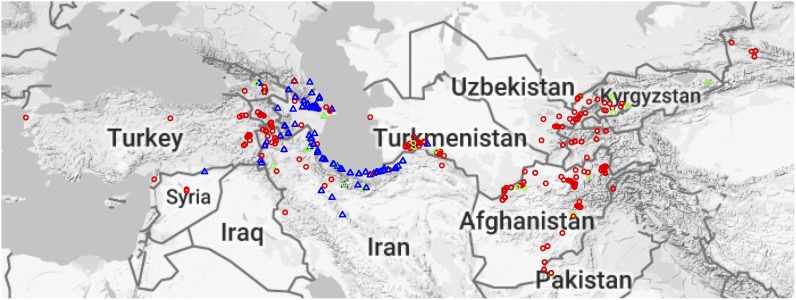
Geographical distribution of *Aegilops tauschii* accessions. Red circles represent Lineage 1 (L1), blue triangles Lineage 2 (L2), and gold plus sign (+) are putative hybrids. Green circles and triangles represent MiniCore accessions, and their shapes represent their lineage.

This study included 569 *Ae. tauschii* accessions from Wheat Genetics Resource Center (WGRC) at Kansas State University (K-State) in Manhattan, KS, United States. Most of the *Ae. tauschii* accessions were collected in 1950s and 1960s from 15 different countries by several explorers, however, a recent exploration was carried out by WGRC scientists in 2012 in Azerbaijan to fill the geographical gaps in the collection and sample more genetic diversity (Supplementary Figure S1 and Supplementary Table S1). Passport data, including longitude and latitude of the collection site, were available for most of the accessions and were plotted on the map to visualize the distribution (Figure 1). To study the relationship between *Ae. tauschii* and hexaploid wheat (*T. aestivum* L.), 103 wheat varieties from a panel of diverse United States winter wheat accessions were also included in the study (Grogan et al., 2016) (Supplementary Table S1).

### Plant Tissue Collection and Genotyping-by-Sequencing

A single plant for each accession was grown in 2″ × 2″ pots in the greenhouse. About five centimeter of leaf tissue from single 2–3 weeks old seedlings were collected in 96-well tissue collection box and stored at −80°C until DNA extraction. Tissues were lyophilized in the lab for 24–36 h, followed by genomic DNA extraction using Qiagen BioSprint 96 DNA Plant Kit (QIAGEN, Hilden, Germany). Extracted DNA was quantified with Quant-iT^TM^ PicoGreen^®^ dsDNA Assay Kit (Thermo Fisher Scientific, Waltham, MA, United States). One random well per plate was left blank for quality control and library integrity. DNA samples were genotyping using genotyping-by-sequencing (GBS) (Poland et al., 2012a). GBS libraries were prepared in 96 plexing using two restriction enzymes—a rare cutter *PstI* (5′-CTGCAG-3′), and a frequent cutter *MspI* (5′-CCGG-3′) with a common reverse adapter ligated. Full protocol is available at the KSU Wheat Genetics website^1^. GBS libraries were sequenced on 10 lanes on Illumina HiSeq2000 (Illumina, San Diego, CA, United States) platform at University of Missouri (UMC; Columbia, Missouri) or McGill University-Génome Quebec Innovation Centre (Montreal, Canada) facility.

### SNP Genotyping and Data Filtering

Single nucleotide polymorphisms (SNPs) discovery and genotyping was performed in single step with Tassel 5 GBSv2 pipeline (Glaubitz et al., 2014), using *Ae. tauschii* genome assembly (Aet v4.0; NCBI BioProject PRJNA341983) as the reference. Tassel was run with *bowtie2* aligner for tags mapping in Linux HPC environment via shell script. Genotypic data were processed in R statistical programming language (R Core Team, 2015) using custom R scripts. Population level SNP filtering was performed and SNPs with minor allele frequency (MAF) less than 0.01 and missing data more than 20% were removed. Further, SNPs with heterozygosity greater than 5% were removed because *Ae. tauschii* accessions are highly inbred. Fisher’s exact test at alpha 0.001 with Bonferroni correction was performed to determine if the putative SNPs were from allelic tags as described in Poland et al. (2012b). Individual samples with more than 80% missing SNP calls and more than 5% heterozygosity were also removed. Retained markers and samples were used for further analyses.

### Population Structure and Ancestry Analysis

Population structure and ancestry analysis was performed with fastSTRUCTURE software (Raj et al., 2014), cluster analysis, and principal component analysis (PCA). Initially, fastSTRUCTURE was run with all filtered SNPs at *K* = 2 using ‘simple’ prior to partition all *Ae. tauschii* accessions into L1 and L2 lineages. Per the developer recommendation for computational efficiency, fastSTRUCTURE was run with ‘simple’ prior and random seed for *K* = 2 to *K* = 8 with three replications each to detect the optimum values of *K*. Once the optimum *K* was determined, final fastSTRUCTURE analysis was performed using ‘logistic’ prior with all the SNPs. Only those accessions with available passport information were used in this analysis, and passport information was used to group and order accessions. To ensure the label collinearity for multiple iterations of each *K* run, fastSTRUCTURE results were processed using *CLUMPAK* package (Jakobsson and Rosenberg, 2007; Kopelman et al., 2015) and plotted using *Distruct* program (Rosenberg, 2004). Optimal *K*-value was determined using ‘chooseK’ utility provided with fastSTRUCTURE.

Phylogenetic cluster analysis was performed in R language. Genetic distances were computed using ‘dist’ function with Euclidean method. Distance matrix was converted to a phylo object using ‘ape’ package (Paradis et al., 2004). Using ‘phyclust’ package (Chen, 2011), a neighbor joining unrooted tree was plotted to indicate subpopulation clusters and identify tentative cryptic outliers that were not identified phenotypically. Cluster analysis was performed using default parameters in ‘dist,’ ‘ape,’ and ‘phyclust.’

Principal component analysis was performed in R language. Eigenvalues and eigenvectors were computed with ‘e’ function using ‘A’ matrix output of rrBLUP package (Endelman, 2011). First three eigenvectors were plotted as three principal components to observe clustering. All analyses were performed separately for *Ae. tauschii* only to detect subpopulation, and with wheat to study the wheat-*Ae. tauschii* relationship. L1 and L2 accessions were identified from fastSTRUCTURE partitioning of two lineages at *K* = 2 and projected onto the PCA. To find the best variables explaining the differentiation within lineages, correlation coefficients were computed for PC2 and PC3 vs. longitude, latitude and altitude.

### Genetic Diversity Analysis

As a measure of average heterozygosity over multiple SNPs in a given population, Nei’s diversity index (Nei, 1973) was computed for the whole population, and separately for L1, L2, wheat, and combined for L1 and L2. Additionally, pairwise *F*_ST_ between subpopulations, and lineage wise minor allele frequency (MAF) were computed and plotted using custom R scripts. Pairwise *F*_ST_ were computed among L1, L2, and wheat in all combinations. MAF plots were plotted separately for L1 and L2.

### Lineage-Specific Allelic Contribution to Putative L1–L2 Hybrids and Wheat D-Genome

Lineage specific private alleles are the ones that are segregating in one lineage but fixed in the other. To determine a lineage specific allele at a SNP site, dataset was split into L1 and L2 accessions. SNP sites where MAF was zero in one lineage but greater than zero in the other lineage, were filtered and the segregating lineage specific allele identified. L1 and L2 private alleles were assigned different colors and plotted for each putative hybrid separately. For each hybrid, lineage specific contribution was determined as percentage of alleles contributed by specific lineage. Using private allele SNPs, allele matching was performed as described in Singh et al. (2019) to find the putative parents of each hybrid from both L1 and L2. For wheat D-genome, a consensus of lineage specific alleles was determined, and lineage specific alleles were plotted across all wheat D-genome chromosomes. For those SNP sites, where more than one wheat lines had L1 specific allele, it was considered as a putative introgression from L1. Lineage specific contribution was determined as percentage of alleles contributed by specific lineage across the consensus.

### Genetically Diverse Representative Core-Set Selection

All SNPs were used to select a representative core-set from the *Ae. tauschii* collection. The core-set was selected in two steps. First, software package PowerCore was used with default settings (Kim et al., 2007), which selects the lines to retain most diverse alleles by implementing advanced M (maximization) strategy. Then the number of selected accessions was further reduced by phenotypically guided selection using the available phenotypic data for Leaf rust composite, Stem rust race TTKSK (Rouse et al., 2011) and Hessian fly biotype D resistance. The diversity captured by the MiniCore was assessed by the percent segregating SNPs present in the selected accessions relative to the whole collection.

## Results

### Geographical Distribution of *Ae. tauschii*

*Aegilops tauschii* is mainly found around the Caspian Sea and in central Asia but is found as far West as Turkey (Lon: 26.327362, Lat: 40.009735) and as far East as eastern China (Lon: 111.048058, Lat: 34.059486). Geographical origin data was known for most of the accessions (Figure 1). The majority of the accessions come from Afghanistan, Iran and Azerbaijan (Supplementary Figure S2). L1 is spread across the entire *Ae. tauschii* geographical range, whereas L2 is only present in Transcaucasia and around the Caspian Sea region (Figure 1). However, we did find one L2 accession in Uzbekistan, which is the first report of an L2 accession out of their natural habitat.

### Genomic Profiling

Genotyping-by-sequencing (GBS) generated 318,639 putative single nucleotide polymorphisms (SNPs) from a total of 672 samples consisting of 569 *Ae. tauschii* and 103 wheat lines. Filtering the SNPs based on missing data, MAF, heterozygosity, and Fisher’s exact test resulted in 13,582 SNPs. Additionally, poor samples were removed based on the amount of missing data and heterozygosity. Twenty *Ae. tauschii* samples with more than 80% missing SNP calls and 5% heterozygosity were removed, which resulted in a dataset of 13,582 SNPs for 652 samples consisting of 549 *Ae. tauschii* and 103 wheat samples. Finally, after removing 447 SNPs that were private to wheat, a total of 13,135 high quality SNPs were retained and used for further analyses.

### Population Structure Analysis

**FIGURE 2:**
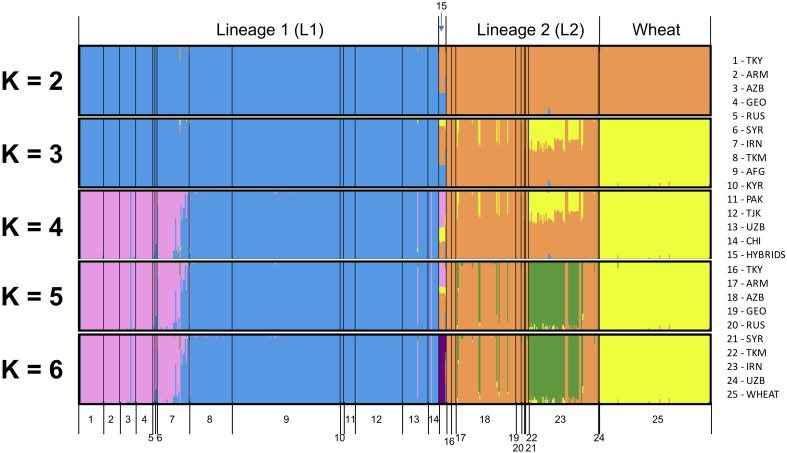
Global population structure analysis for *Ae. tauschii* L1, L2, putative hybrids and wheat for *K* = 2 to *K* = 6. An additional color is added with each increase in the value of *K*. Each vertical bar represents an individual, where the proportion of the color bar representing membership coefficient for each subpopulation. A bar with only a single color represents its ancestry to a single population, and a mixture of colors represents admixture from different populations.

All SNPs were used to infer the ancestry of filtered samples using variational Bayesian inference algorithm fastSTRUCTURE. Global analysis was run for *Ae. tauschii* and wheat together for *K* ranging from two to eight with three iterations for each *K* (Figure 2). Samples were pre-assigned labels based on their geographical origin, and this information was used for plotting the membership coefficients. At *K* = 2, L1 and L2 split from each other within *Ae. tauschii* and wheat remained clustered with L2 of *Ae. tauschii*. Nine accessions showed a very distinct structural differentiation as admixture of L1 and L2 (Figure 2; group 15). These nine accessions were hypothesized as the possible hybrids between L1 and L2 and were analyzed separately. Using “chooseK” utility provided with fastSTRUCTURE *K* = 6 was determined to be the optimal, where marginal likelihood of the data was maximized. For this study, we also found that *K*-values ranging from 2 to 6 were optimal and gave biological and geographic inference. At *K* = 3 L1, L2 and wheat were completely separated, with over half of the Iranian and few Azerbaijan accessions from lower altitudes showing admixture. At *K* = 4, L1 showed sub-population differentiation where accessions from Armenia, Azerbaijan, Georgia, Russia, Syria, and Turkey clustered separately from accessions originated in Afghanistan, China, Kyrgyzstan, Pakistan, Tajikistan, Turkmenistan, and Uzbekistan. Accessions from Iran showed mixture of accessions from these two groups. Putative hybrids showed clear similarity with accessions from the western side of Caspian Sea in L1 and Iranian accessions in L2. At *K* = 5, L2 accessions showed some differentiation where more than half of the accessions from Iran occurring at lower altitudes differentiated from Armenia, Azerbaijan, Georgia, and Turkey. For *K* > 5 no further information was provided by the population structure analysis in terms of population differentiation within L1 and L2, however, putative hybrids formed their own cluster. Wheat showed no sub-population differentiation at all. Therefore, we determined *K* = 5 to be a secondarily optimal stratification level after the optimal *K* = 3.

Population structure analysis was also run only on *Ae. tauschii* to determine the impact of the wheat outgroup on the pattern of *Ae. tauschii* grouping (Supplementary Figure S3). Marginal likelihood of the data was maximized at *K* = 5. At *K* = 2, L1 and L2 differentiated strongly, and the same group of nine accessions as possible hybrid was evidenced as admixture of L1 and L2. At *K* = 3, L1 showed the same population differentiation as the global analysis. Accessions from the eastern side of Caspian Sea differentiated from the western side. At *K* = 4, L2 Iranian accessions showed admixture and differentiate from other accessions. At *K* = 5, putative hybrids differentiated to form their own cluster. At *K* > 5 no more useful information was provided by the population structure analysis.

### Principal Component and Cluster Analysis

**FIGURE 3:**
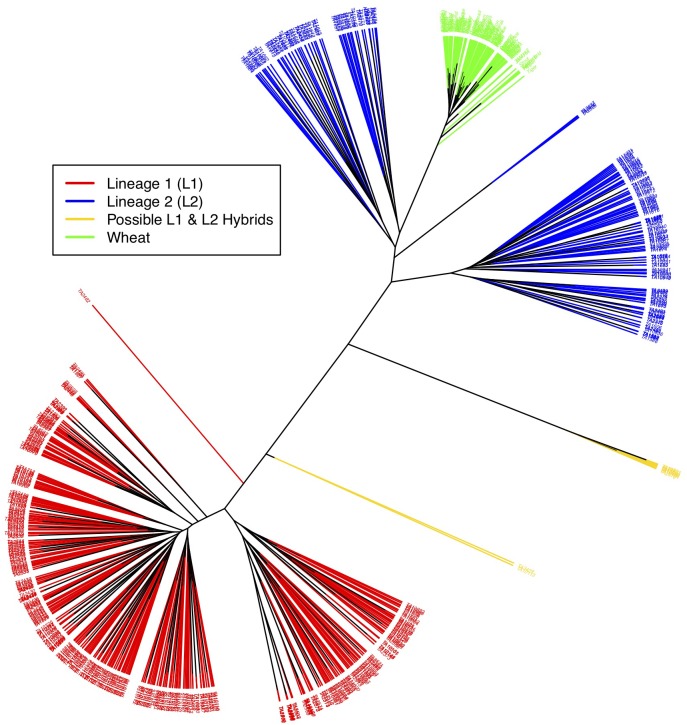
Neighbor-joining tree showing relationship between L1, L2, possible L1–L2 hybrids and wheat. Red branches represent L1 accessions, blue L2, gold L1–L2 hybrids, and green wheat. Wheat is closely related to L2 of *Ae. tauschii*. Putative hybrids cluster out separately and appear in between the two lineages.

Principal component analysis was run as a second approach to cluster accessions and detect subpopulations. The same set of 13,135 *Ae. tauschii* specific SNPs were used for PCA. The inferred lineages for *Ae. tauschii* individuals by population structure analysis were used to color the accessions in PCA (Supplementary Figure S4) and phylogenetic cluster analysis (Figure 3). Principal component analysis was performed separately for two datasets- *Ae. tauschii* with wheat, and *Ae. tauschii* only. As expected, the population differentiation observed by fastSTRUCTURE was confirmed with PCA as three distinct groups–L1, L2 and wheat–were observed in the first two components of the PCA (Supplementary Figure S4). PC1 explained 55% of the variation separating L1 and L2. PC2 explained 7% of the variation and separates out wheat from L2 of *Ae. tauschii*. Corroborating previous reports, the wheat was observed to be more closely related to L2 accessions.

**FIGURE 4:**
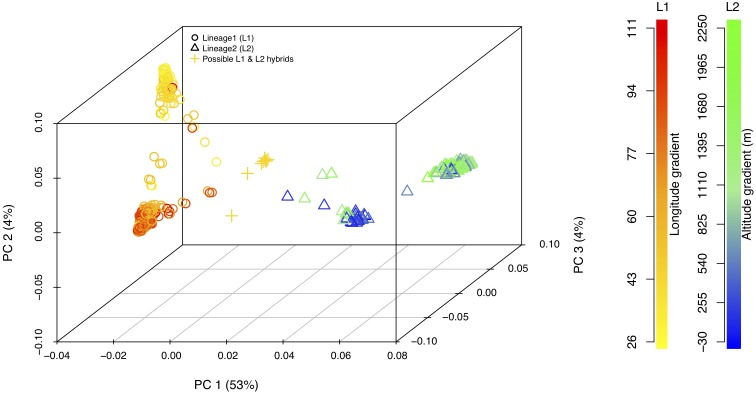
3-D principal component analysis plot for L1, L2, and possible L1–L2 hybrids. Lineage1 (L1) is colored based on the longitudinal gradient and Lineage2 (L2) is colored with altitudinal gradient with reference to the sea level. Empty circles represent L1 (yellow–red gradient) and empty triangles represent L2 (blue–green gradient). Gold plus sign (+) represent putative L1–L2 hybrids. Legends for the color gradient are shown on the right-hand side.

**FIGURE 5:**
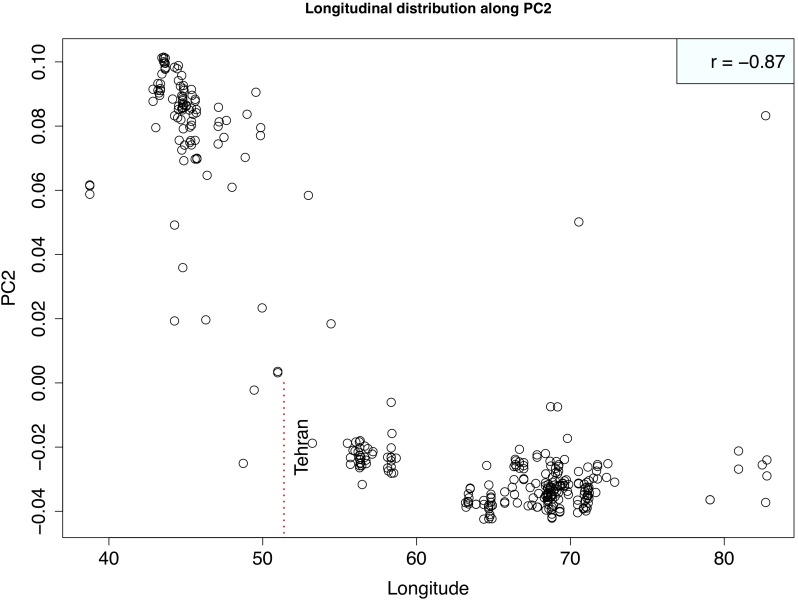
Scatterplot showing the relationship between Longitude of Lineage1 accessions and second principal component. Strong correlation between two variables is evident suggesting that PC2 is separating out western (lower longitudes) from eastern (higher longitudes) accessions. Correlation coefficient is shown at the top right corner. Vertical red dotted line marks the longitude of Tehran, Iran that demarcates the eastern vs. western accessions.

Principal component analysis with only the *Ae. tauschii* accessions, also confirmed the strong population differentiation between two *Ae. tauschii* lineages, L1 and L2. In this analysis, PC1 explained 53% the variation in the dataset (Figure 4 and Supplementary Figure S4). When analyzed in the absence of wheat, L1 shows a strong within lineage differentiation on the second principal component explaining 4% of the variation, and L2 on the third principal component explaining 4% of the variation (Figure 4). To find the variables explaining the most variation within lineages along PC2 and PC3, the correlation coefficients were computed to agroclimatic variables. Correlation analysis showed that the L1 differentiation was strongly correlated with the longitudinal gradient of accessions with an east-west gradient relative to the Caspian Sea, and L2 with altitude relative to sea level (Supplementary Figure S5). After removing the outlier accessions, when the longitudes of L1 accessions are plotted against PC2, it clearly separated the accessions in east and west of Tehran, Iran (Figure 5). On the third principal component, population differentiation was also observed, which corresponded to the altitude of origin of the L2 accessions in reference to the sea level (*r* = 0.61). PC3 vs. altitude plot also shows a clear trend with PC3 separating the accessions according to their altitude, however, there are few outliers present on the both ends (Supplementary Figure S6). Generally, lower altitude accessions clustered together separately from the higher altitude accessions. We found that the strongest differentiation between L2 clusters was at around 150m above sea level. Overall the PCA results were in strong agreement with the population differentiation observed with fastSTRUCTURE.

As a final assessment of population structure, Cluster analysis was performed by computing genetic distances among accession using Euclidean method. An unrooted tree in this cluster analysis splits samples into three distinct clades- L1, L2 and wheat (Figure 3). Wheat and L2 were more closely related than wheat and L1, and L1 and L2. L1 and L2 further shows two clades within that could again be attributed to longitudinal variation from the Caspian Sea and altitude, respectively. Wheat essentially did not show any differentiation within.

### Admixed *Ae. tauschii* Accessions Are L1–L2 Hybrids, or Possibly a New Lineage

**FIGURE 6:**
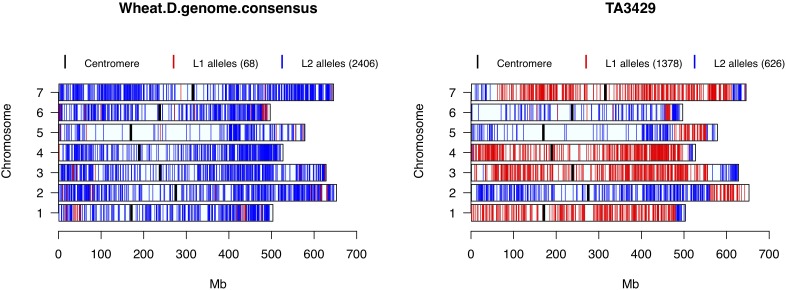
Wheat D-genome consensus and putative hybrid TA3429 chromosomes showing the distribution of L1 and L2 specific alleles. Red color represents L1 specific alleles, and blue represents L2 specific alleles. Centromeres are marked with thick black bars. Numbers in parentheses in the legend represent the number of lineage specific alleles.

Nine accessions showed up in STRUCTURE, PCA and cluster analysis as admixture of *Ae. tauschii* lineages L1 and L2. To test their origin as hybrids between L1 and L2 accessions, private alleles in both lineages were filtered and tested in the hybrid samples. A total of 4,711 L1 and 4,700 L2 private alleles were identified in the whole collection. Based on the total number of SNPs assayed in putative hybrids, lineage specific alleles contributed by L1 ranged from 48 to 70%, and L2 ranged from 30 to 52%. Out of nine putative hybrid samples, only TA3429 was confirmed as a typical bi-parental recombinant inbred line between L1 and L2 accession(s), in which the chromosomal segments from L1 and L2 were clearly demarcated without any overlap (Figure 6). The other eight putative hybrids showed no such clear pattern but ambiguous distribution of private alleles (Supplementary Figure S7). Private alleles were visualized for one randomly selected accession from each L1 and L2, which showed no contribution from the other lineage (top row, Supplementary Figure S7).

Seven out of the eight unclear putative hybrids originated in Georgia and one in Turkmenistan. A total of 2,098 SNPs with private L1 or L2 alleles were assayed in these hybrids. Exploring further, we found that 1,988 SNPs were fixed (private alleles contributed by L1 or L2). Only 110 SNPs were segregating mostly from the one accession from Turkmenistan (Supplementary Figure S8). Removing that accession left only six segregating SNPs and 1,768 fixed SNPs in seven putative hybrids from Georgia. Failing to construct their expected hybrid haplotypes, we hypothesized that these putative hybrids from Georgia are an isolated lineage that probably resulted from a single hybridization event between an L1 and L2 accession. To determine this, we filtered out the SNPs to find if there were any alleles private to these hybrids, and we found 713 SNPs with alleles private to these hybrids from Georgia. Of these 713 SNPs, only 29 were segregating within these hybrids, and the remaining were fixed.

To find potential L1 and L2 parents of each putative hybrid, allele matching was performed. SNPs with lineage specific private alleles were used to find the closest accession from each lineage. Lowest and highest percent identity was found to be 76.96 and 85.02%, respectively, between a pair of hybrid and L1 accessions. Similarly, the lowest and highest percent identity between any pair of hybrid and L2 accessions was found to be 74.2 and 77.62%, respectively. These lower identity coefficients confirm that the potential parental accessions of these putative hybrids were not found in this collection. List of putative hybrids with highest matching accessions is summarized in Table 1.

**Table 1:** Putative hybrids and their tentative L1 and L2 parents.

Putative hybrid	Origin	Putative L1 parent	Putative L2 parent
TA10103	Georgia	TA10181 (76.96%)	TA2527 (76.78%)
TA10104	Georgia	TA10181 (77.3%)	TA2527 (77.13%)
TA10113	Turkmenistan	TA10181 (83.65%)	TA2527 (73.64%)
TA10928	Georgia	TA10181 (77.43%)	TA2527 (77.62%)
TA10929	Georgia	TA10181 (77.2%)	TA2527 (77.14%)
TA2576	Georgia	TA10181 (77.24%)	TA2527 (77.49%)
TA2580	Georgia	TA10181 (77.17%)	TA2527 (76.93%)
TA2582	Georgia	TA10181 (77.47%)	TA2527 (76.92%)
TA3429	Unknown	TA1634 (85.02%)	TA2377 (74.2%)

*Numbers in parenthesis represent percent identity of the hybrid with the parent.*

### Lineage-Specific Private Allelic Contribution to Wheat D-Genome

All wheat lines had similar distribution of lineage specific alleles across all chromosomes with minor differences (data not shown), therefore, to determine the lineage-specific contribution of *Ae. tauschii* to wheat D-genome, a consensus of private alleles distribution was determined. As it has been shown that the L2 of *Ae. tauschii* contributed the D-genome of wheat, all private alleles were assumed to be contributed by the L2. However, if at least two different wheat lines carried the same private allele from L1 at a given SNP site, it was considered a putative introgression from L1 in the consensus. We observed that the D-genome consensus carried only 68 (2.7%) alleles from L1 and 2,406 (97.3%) alleles from L2. Two chromosomes, 1D and 6D, carried 54% of the total L1 alleles with majority of the introgressions present in the distal regions (Figure 6).

### Genetic Diversity

Nei’s diversity index was computed using all SNPs separately for *Ae. tauschii* L1, L2, possible hybrids, wheat and *Ae. tauschii* collection combined. Highest Nei’s diversity index was observed for L2 = 0.1326 followed by L1 = 0.0872, and wheat of 0.0158. Higher values of the Nei’s index indicates greater allelic diversity in a given population. Combined Nei’s index for *Ae. tauschii* was 0.2382 and the whole dataset including wheat was 0.2597.

To evaluate population differentiation between the different pairs of *Ae. tauschii* lineages and wheat, pairwise *F*_ST_ statistics were computed. Highest F_ST_ were observed between L1 and wheat, followed by wheat and L1–L2 hybrids, and wheat and L2 (Table 2). The population differentiation between L1 and wheat also supports the large number of novel of alleles found in this lineage that are absent from the wheat pool.

**Table 2:** Pairwise *F*_ST_ coefficients among L1, L2, L1–L2 hybrids, and Wheat.

	L2	L1–L2	Wheat
L1	0.5635	0.0928	0.6261
L2	–	0.1183	0.3046
L1–L2	–	–	0.5262

*Higher values represent stronger population differentiation.*

Minor allele frequency was computed and plotted separately for L1, L2 and jointly for both lineages (Supplementary Figures S9, S10). Individually, MAF spectrum for L1 and L2 showed an expected distribution with majority of alleles present at very low frequency (Supplementary Figures S9A,B). Joint distribution of L1 and L2 MAF revealed that majority of the alleles segregating in one lineage were close to fixation in the other lineage (Supplementary Figure S9C). Chromosome-wise map for MAF, revealed that majority of the polymorphic markers were present on the distal ends of the chromosomes (Supplementary Figure S10), and L2 has higher proportion of polymorphic markers as indicated by the density and height of L2 bars.

### Core-Set Selection

Genetically diverse core-set was selected using software package PowerCore that implements advanced M (maximization) strategy to select diverse accession by reducing allelic redundancy and keeping the allele frequency spectrum similar. Initially 107 *Ae. tauschii* accessions were selected using advanced M strategy implemented in PowerCore (Supplementary Table S2). These accessions were then plotted on a phylogenetic tree and selected using known phenotypic information on disease and insect resistance to get the size of this core to a manageable number. This selection was guided by phenotypic data for resistance to Leaf rust composite, Stem rust TTKSK race and Hessian fly biotype D. Other factors, such as the available geographical origin and the history of their previous use in genetic mapping, were also taken into account to pick the representative accessions. Finally, 40 accessions were selected to comprise a MiniCore that is distributed uniformly across the WGRC *Ae. tauschii* collection (Supplementary Figure S11). Nei’s diversity index computed for the MiniCore (0.2235) compared to the whole collection (0.2382) suggests allelic richness in the MiniCore. Also, in the MiniCore, we were able to retain the 11,041 segregating SNPs out of 13,135 from the whole *Ae. tauschii* collection. By reducing the collection size by over 10-fold, we were still able to capture ∼84% of the segregating alleles present in the whole WGRC collection. MiniCore consists of 29 accessions from L1 and 11 accessions from L2 of *Ae. tauschii*.

## Discussion

### Geographical Distribution of *Ae. tauschii*

Caspian Sea region is thought to be the center of origin of *Ae. tauschii*. Most of the accessions in our collection were also sampled from this region (Figure 1). Consistent with the current literature, we observed in our study that L2 of *Ae. tauschii* is spread on a narrow longitudinal range from northeastern Syria to northeastern Iran spanning a distance of 1625 km, whereas L1 is found from southern Turkey to northwestern China, spanning over 4000 km. However, we did find one L2 accession TA10124 originated in Uzbekistan. It is possible that the passport data for this accession was recorded wrong, but if true, it might point to the possibility of L2 migrating out of its natural habitat and extending eastbound. However, more sampling is required to make any further claims. Most of the accessions were acquired from other genebanks, however, to fill up the geographical gaps, a recent exploration was conducted in 2012 by WGRC researchers (blue dots, Supplementary Figure S1). Multiple accessions from both lineages are found to overlap at similar altitudes, with L1 accessions generally inhabiting higher altitudes than L2 (Supplementary Figure S12A). Majority of L1 and L2 accessions fall in the similar latitude distribution, but some L1 accessions were widely spread (Supplementary Figure S12C).

### SNP Discovery and Ascertainment Bias

Using *Ae. tauschii* genome assembly Aet v4.0 as the reference, GBS produced 13,135 high quality SNP markers useful to assess genetic diversity in the collection. We expected some bias in the two lineages because the reference genome (Aet v4.0) represents *Ae. tauschii* ssp. *strangulata*. However, as we did not use any prior SNP information to call SNPs, we expect the ascertainment bias be minimal. Splitting two lineages and computing MAF separately revealed that both lineages had about similar distribution of MAF (Supplementary Figure S9), but with elevated MAF in L2 (Supplementary Figure S10). Because the goal of this project was not to assess any specific genomic region, using Aet v4.0 reference genome should not pose a problem.

### Population Structure Analysis

Global population structure analysis showed the expected *Ae. tauschii* subpopulations (L1 and L2) with each having two subgroups, and wheat D-genome forming a third group which was most closely clustered to L2. These findings are largely in agreement with known population structure of *Ae. tauschii*, confirming the utility of our genotyping approach. In addition to these five groups, we unexpectedly found putative hybrids clustered together (Figure 2). This small group of nine accessions showed up as admixture of L1 and L2. At *K* = 3 wheat split from L2 sharing ancestry with most of Iranian and few Azerbaijan accessions. One common feature of these accessions is that they all occur at lower altitudes from the sea level. This points to the possibility that these accessions or their ancestors could have been involved in the origin of wheat.

*Aegilops tauschii* L1 showed intra-lineage population differentiation in accordance with relative position of East or West of the Caspian Sea. This was also clear in the principal component analysis where L1 was differentiated by PC2 along longitudinal gradients (Figure 4). Iranian accessions did not show clear population differentiation by falling into the eastern or western group but rather show admixture. Iran is at the center of origin for *Ae. tauschii* and could be seen as a transition region for the East and West clades of L1. The majority of the L2 accessions occur in Azerbaijan and Iran, both of which are on one side of the Caspian Sea with Iran expanding to the eastern side, therefore longitudinal gradient did not explain much of the weak population structure within L2 at *K* = 5. However, we found that this population differentiation could be attributed to the altitude of the origin of L2 accessions where accessions originating at less than 150 m above sea level cluster separately from the accessions from more than 150 m above sea level (Supplementary Figure S6).

The accessions that were admixture clustered separately from all other accessions and did show unique ancestry. These admixed putative hybrids were observed to have shared ancestry with L1 accessions from Turkey and Transcaucasia, and L2 accessions from Iranian and Azerbaijan accessions occurring at lower altitudes. This could possibly mean that their original parents belong to these geographical regions.

### Inter-Lineage Hybridization and the Origin of a New Lineage

*Aegilops tauschii* is a highly self-pollinated species, therefore natural hybrids between L1 and L2 are rare and have been the subject of limited reports. Wang et al. (2013) reviewed that collectively, only 1.4% accessions have been classified L1–L2 intermediates in several studies. They also found two intermediate accessions falling in between L1 and L2. Based on haplotype distribution similarity and close geographical proximity of origin, they speculated that these two accessions could have originated from the hybridization of a single L2 plant with an L1 plant.

In the present study, we found nine such intermediate accessions that fall in between the L1 and L2 in the fastSTRUCTURE, PCA and cluster analyses. Using the SNPs with private alleles, the allele matching of putative hybrids with L1 and L2 accessions did not result in a perfect match, which suggests that the real parental accessions could be missing in our collection. This suggests that the natural hybridization of L1 and L2 accessions is indeed rare, and these hybrids possibly originated from one or few of these rare events. These findings are in alignment with Wang et al. (2013), where they suggested a single hybridization event could have resulted in the two intermediate individuals in their data. Seven of the hybrids identified in our study were found in Georgia, one in Turkmenistan, and one with missing passport data. Both lineages co-exist in Georgia and Turkmenistan, therefore they are not isolated by distance. It is possible that they are reproductively isolated given their inbreeding nature. Similar pattern of reproductive isolation and rare hybridization was reported in rice landraces (Huang et al., 2010), and switchgrass (Mizuno et al., 2010; Sohail et al., 2012; Grabowski et al., 2014).

The distribution of L1 and L2 private alleles in these admixed accessions supports our hypothesis that these accessions could have arisen from L1 to L2 hybrids (Figure 6 and Supplementary Figure S7). One hybrid, TA3429, showed a typical recombinant inbred pattern, which was different than other hybrids. This accession was actually received from a Japanese collection with few other germplasm lines, and was labeled as 4× (tetraploid). However, when tested phenotypically and cytologically, it was diploid like normal *Ae. tauschii*. Therefore, it is possible that this accession was in fact an artificially created hybrid between an L1 and L2 accession as a diploid.

All other admixed accessions appear to be derived from a rare hybridization event between an L1 and L2 accession followed by isolation and possibly multiple intercrossing events. We found that the majority of the L1, L2 private SNP alleles assayed in these putative hybrids were fixed, and only 110 were segregating with majority of the hybrids carrying same private alleles. Exploring further, we identified 713 SNPs with alleles private to the admixed accessions from Georgia. Of these 713 SNPs, only 29 were segregating among these hybrids. Together this supports the possibility that these accessions resulted from single hybridization event. Though a limited sample, this points to the possibility of development of an unreported lineage as a result of rare hybridization event between an L1 and L2 accession, however, more samples are needed from these areas to shed new light on the nature of hybridizations among both lineages.

### Genetic Diversity

Wheat had the lowest Nei’s index, which is expected because of its domestication and polyploidization, compared to its wild progenitor, *Ae. tauschii*. Reduction in genetic diversity has also been reported in cotton as a result of change in ploidy level (Iqbal et al., 2001). Wheat lines in our study also represent a relatively narrow collection of United States winter wheat, leading to the lowest Nei’s index. Highest Nei’s index was observed for L2, followed by L1. This can be attributed to the differences in distribution of L1 and L2 across their natural habitat. L1 is distributed across the longitudinal gradient, whereas L2 is distributed across the altitudinal gradient. Latitude is known to affect the weather temperature with cooler temperatures away from the equator (Rind, 1998), but the latitude distribution for L1 and L2 was similar for the majority of accessions except few outliers (Supplementary Figure S12C). Therefore, the expected effect of latitude should be minimal. Longitude distribution for L1 was more extensive as compared to L2 (Supplementary Figure S12B). As shown in Figure 1, the majority of the L2 accessions are distributed around the Caspian Sea as compared to very few L1 accessions. Therefore, the longitude effect is more pronounced in L1 than L2. Moreover, the altitude distributions for L1 and L2 were also different (Supplementary Figure S12A), with more L2 accessions growing at lower altitude. Altitude is known to have an effect on the temperature (Körner, 2007). Therefore, L2 accessions might have selected alleles to survive in different temperatures. Combined *Ae. tauschii* had higher Nei’s index as compared to any single lineage, which is expected because all the allelic diversity is assayed in the whole collection.

### *Ae. tauschii* Contribution to the Wheat D-Genome

We assayed wheat D-genome chromosomes for lineage specific introgressions from *Ae. tauschii*. A majority of the introgressions mapped to L2, which is consistent with the current and past literature. Calculating the percentage of lineage specific alleles, we observed that L1 had only contributed 2.7% of the total *Ae. tauschii* introgressions in comparison to 97.3% by L2. This supports previous reports and points to the need to use L1 accessions for broadening the genetic base of hexaploid wheat and harness the untapped genetic diversity present in *Ae. tauschii* L1. With this goal, we developed a small set of *Ae. tauschii* (MiniCore) consisting of 29 L1 and 11 L2 accessions to facilitate wheat improvement.

### Genetically Diverse Representative MiniCore

Accessing the genetic diversity present in wild relatives can be a challenging task for breeders due to the large number of accessions and confounding physiology of the wild plants. Wild accessions with overall poor phenotype could be the source of agronomically important alleles. Efficient use of germplasm collections can often be facilitated through a targeted subset of the total accession that is optimized to capture a maximum amount of the total diversity in a minimum number of accessions. To facilitate the use of *Ae. tauschii* accessions in wheat breeding, we selected only 40 accessions to develop a small MiniCore set that captures 84% of the segregating alleles from the whole collection. MiniCore was carefully selected from both the lineages of *Ae. tauschii* but the main focus was to target more from L1. This is because L1 is a reservoir of untapped genetic diversity that has not been leveraged by the breeders. L2 accessions were chosen because this lineage is the source for many of the diseases and insect resistance. These accessions can be utilized to bring in novel genetic variation for wheat rusts, insect resistance, heat and drought tolerance to produce climate resilient wheat varieties. This MiniCore consisting of genetically diverse accessions was selected with an objective to broaden the genetic base of wheat D-genome. However, in future, the selection can be optimized based on the recombination rate and the distribution of *Ae. tauschii* regions that are already introgressed in the wheat D-genome.

### Future Work and Strategy to Utilize Genetic Diversity in *Ae. tauschii*

**FIGURE 7:**
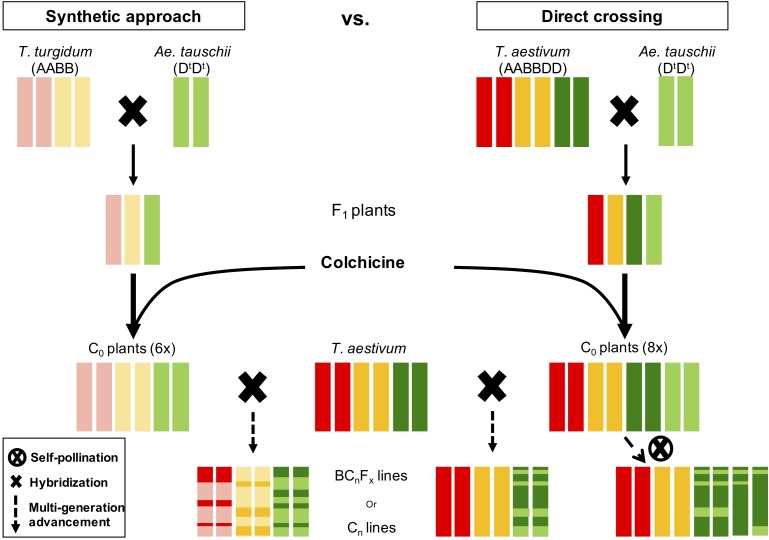
Side-by-side comparison of two strategies to introgress genetic diversity from *Ae. tauschii* to wheat. Red bars represent A-genome, gold B-genome, dark green wheat D-genome, and light green *Ae. tauschii* D-genome. In synthetic approach the resulting line is a mosaic of all three genomes whereas in direct crossing only D-genome is shuffled.

Untapped genetic diversity in *Ae. tauschii* is of great interest to breeders and geneticists for wheat improvement and broadening the narrow D-genome (Kihara, 1944; Lagudah et al., 1991; Lubbers et al., 1991; Akhunov et al., 2010). *Aegilops tauschii* has been utilized via synthetic bridge crossing and direct crossing (McFadden and Sears, 1945; Kihara and Lilienfeld, 1949; Gill and Raupp, 1987), however, both of these strategies have drawbacks. Synthetic bridge crossing involves a tetraploid parent that ultimately brings the genetic diversity in A and B genomes, which makes it difficult and time-consuming process to get rid of undesirable diversity from A and B genomes (Figure 7). Whereas, direct crossing of *Ae. tauschii* with wheat generally result in high F_1_ sterility rendering it less lucrative to researchers (Olson et al., 2013; Cox et al., 2017). However, another novel strategy, which adopts beneficial steps from both these strategies, is “octo-amphiploid bridge” mediated direct genetic transfer, which hasn’t been reported in literature much. Using this strategy, Zhang et al. (2018) recently identified 18 QTLs for three agronomic traits, i.e., thousand kernel weight, spike length, and plant height. Briefly, this strategy involves crossing *Ae. tauschii* directly with wheat producing a haploid F_1_ (*n* = 28; ABDD^t^; Supplementary Figure S13), followed by colchicine doubling resulting in an octo-amphiploid (2*n* = 8*x* = 56; AABBDDD^t^D^t^) (Figure 7). This octoploid can then be either self-fertilized for several generations to develop recombinant inbred lines (RIL) population or backcrossed with hexaploid wheat to develop near isogenic lines for genetic mapping. Since there are four copies of D-genome chromosomes, the progeny will follow tetrasomic inheritance for any given trait with five expected genotypes; nulliplex, simplex, duplex, triplex and quadriplex. Presence of range of genotypes with a single allele differences present an opportunity to study the dosage effect in addition of the genetic mapping. Extending the disomic inheritance model to this octoploid, typical RIL like 96% homozygosity should be achieved after 20 generations of selfing compared to six generations for disomic inheritance (Supplementary Figure S14). However, theoretically moderate frequencies for homozygous individuals for each allele (nulliplex and quadriplex) are achieved at F_5_ or F_6_ that can be used for genetic mapping. Once an associated genetic marker is identified for a trait, it can be used to identify a homozygous line for a given trait and backcrossed with wheat recover euploid wheat (2*n* = 6*x* = 42) with a desired gene introgressed in it (Supplementary Figure S15). Our initial results indicate that depending on the hexaploid wheat used, euploidy can be achieved as soon as after one or two backcrosses.

## Conclusion

Studying genetic diversity in *Ae. tauschii* is very important to wheat improvement in the wake of unpredictable climate and evolving biotic stresses. In this study, we confirmed that *Ae. tauschii* L1 has immense amount of untapped genetic diversity that can be used for wheat improvement. We also provided the evidence of natural *Ae. tauschii* L1–L2 hybrids, which opens the door to the possibility of new genetic variation. Finally, selection of forty genetically diverse accessions will facilitate the use of *Ae. tauschii* for wheat improvement for abiotic and biotic stresses via octo-amphiploid mediated bridge crossing, which will ultimately result in higher genetic gains and faster wheat improvement.

## Data Availability

Sequence reads generated using genotyping-by-sequencing are available from NCBI SRA under accession SRP141206. R-code and other scripts are available at GitHub repository https://github.com/nsinghs/Code_Ae_tauschiiDiversity.

## Author Contributions

NS analyzed the data and wrote the manuscript. SW performed the GBS. VT and SS developed and contributed to the idea. JR acquired, managed, and provided *Ae. tauschii* accessions. DW collected and provided phenotypic data. MA provided *Ae. tauschii* accessions. BG and JP conceived and developed the idea. JP wrote the manuscript.

## Conflict of Interest Statement

The authors declare that the research was conducted in the absence of any commercial or financial relationships that could be construed as a potential conflict of interest.

## Footnotes

1 http://wheatgenetics.org/download/send/3-protocols/74-gbs-protocol
