# Supplementary material for: Genomic Analysis Confirms Population Structure and Identifies Inter-Lineage Hybrids in Aegilops tauschii
Source: Front Plant Sci. 2019 Jan 25;10:9. doi: 10.3389/fpls.2019.00009 (PMC6357674; doi:10.3389/fpls.2019.00009)

Supplementary Material

Genomic analysis confirms population structure and identifies inter-lineage hybrids in *Aegilops tauschii*

Narinder Singh[1], Shuangye Wu[1], Vijay Tiwari[2], Sunish Sehgal[3], John Raupp[1], Duane Wilson[1], Mehraj Abbasov[4], Bikram Gill[1], Jesse Poland[1*]

Following supplementary information is available in this file:

Two tables as additional MS Excel files-
Table S1. List of *Ae. tauschii* accessions and wheat lines with associated metadata.
Table S2. List of *Ae. tauschii* PowerCore and MiniCore accessions with associated metadata.

Fifteen inline figures are inserted in this file-
Figure S1. Geographical distribution of *Ae. tauschii* accessions.
Figure S2. Bar plot showing country-wise contribution of *Ae. tauschii* accessions.
Figure S3. Population structure analysis for only *Ae. tauschii* accessions for K=2 to K=5.
Figure S4. Principal component analysis for *Ae. tauschii* accessions and wheat (upper panel), and *Ae. tauschii* accessions only (lower panel).
Figure S5. Corrplot showing correlation coefficients among different variables and principal component 2 (left), and principal component 3 (right).
Figure S6. Scatterplot showing the relationship between Altitude of Lineage2 accessions and third principal component.
Figure S7. One randomly selected L1 (TA10144) and L2 (TA1664), and putative hybrid accessions' chromosomes showing the distribution of L1 and L2 specific alleles.
Figure S8. Distribution of lineage specific alleles for 110 segregating SNPs.
Figure S9. Minor allele frequency histogram for (A) L1 and (B) L2. (C) joint minor allele frequency scatterplot.
Figure S10. Distribution of minor allele frequencies across all seven chromosomes of *Ae. tauschii*.
Figure S11. Distribution of MiniCore accessions (red branches) in the whole collection.
Figure S12. Violin plots showing L1 and L2 distribution for (A) altitude, (B) longitude, and (C) latitude. Red dots are median values.
Figure S13. Cytological confirmation of wheat x *Ae. tauschii* F_1 hybrid (n=28; $ABDD^t$).
Figure S14. Theoretical change in the genotype frequencies with the advance of generations.
Figure S15. Crossing scheme to retrieve euploid wheat (2n=6x=42).

Figure S1. Geographical distribution of *Ae. tauschii* accessions. Blue dots represent recent 2012 collection.

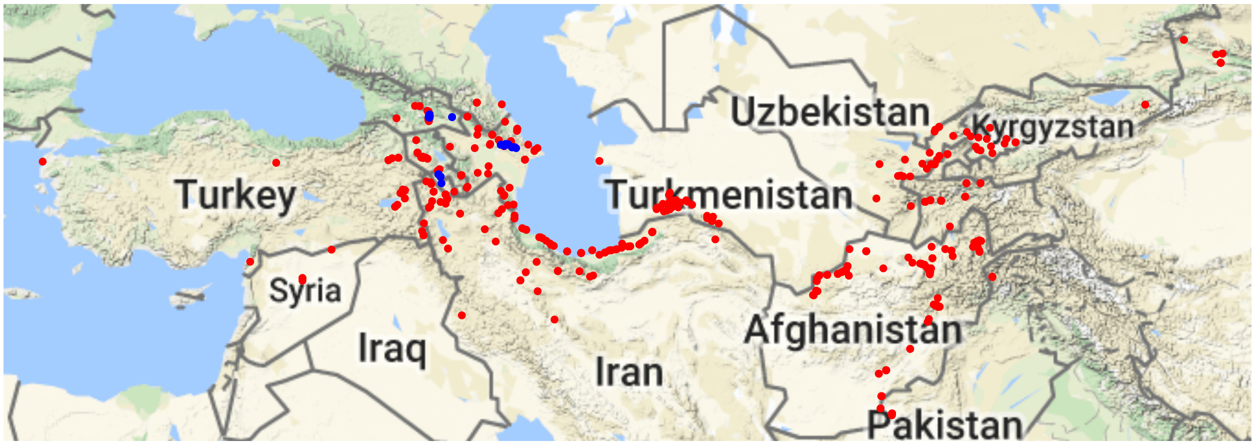

Figure S2. Bar plot showing country-wise contribution of *Ae. tauschii* accessions. Numbers inside the bars represent actual count of accessions.

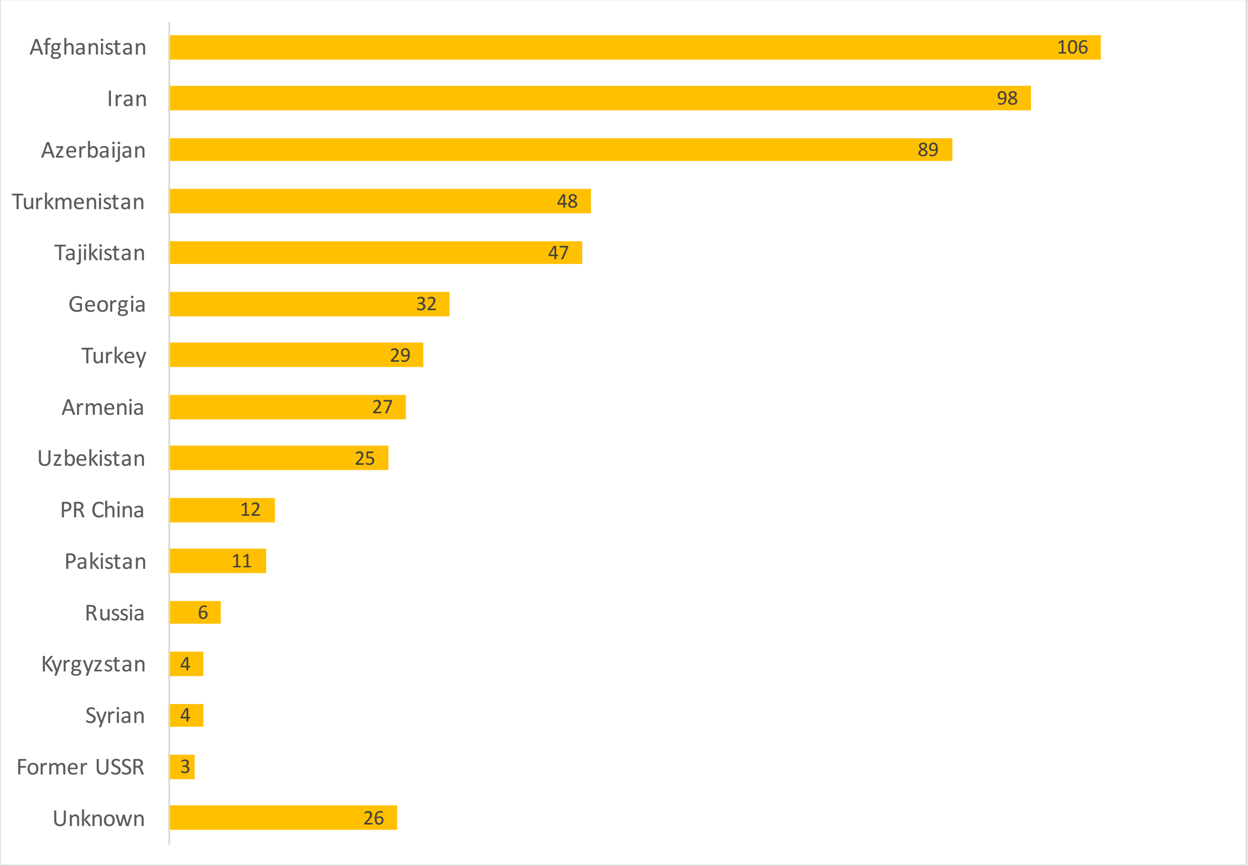

Figure S3. Population structure analysis for only *Ae. tauschii* accessions for K=2 to K=5. An additional color is added with each increase in the value of K. Each vertical bar represents an individual, where the proportion of the color bar representing membership coefficient for each subpopulation. A bar with only a single color represents its ancestry to a single population, and a mixture of colors represents admixture from different populations. Numbers below the plot are the country codes (same as the Fig.2 in the main text).

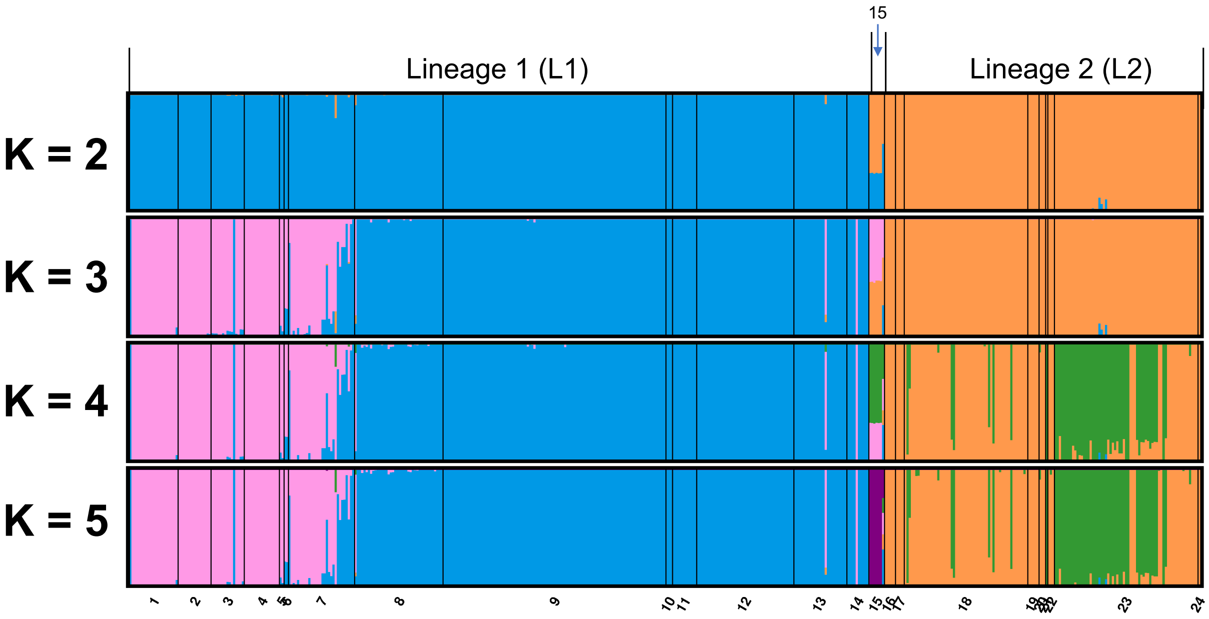

Figure S4. Principal component analysis for *Ae. tauschii* accessions and wheat (upper panel), and *Ae. tauschii* accessions only (lower panel).

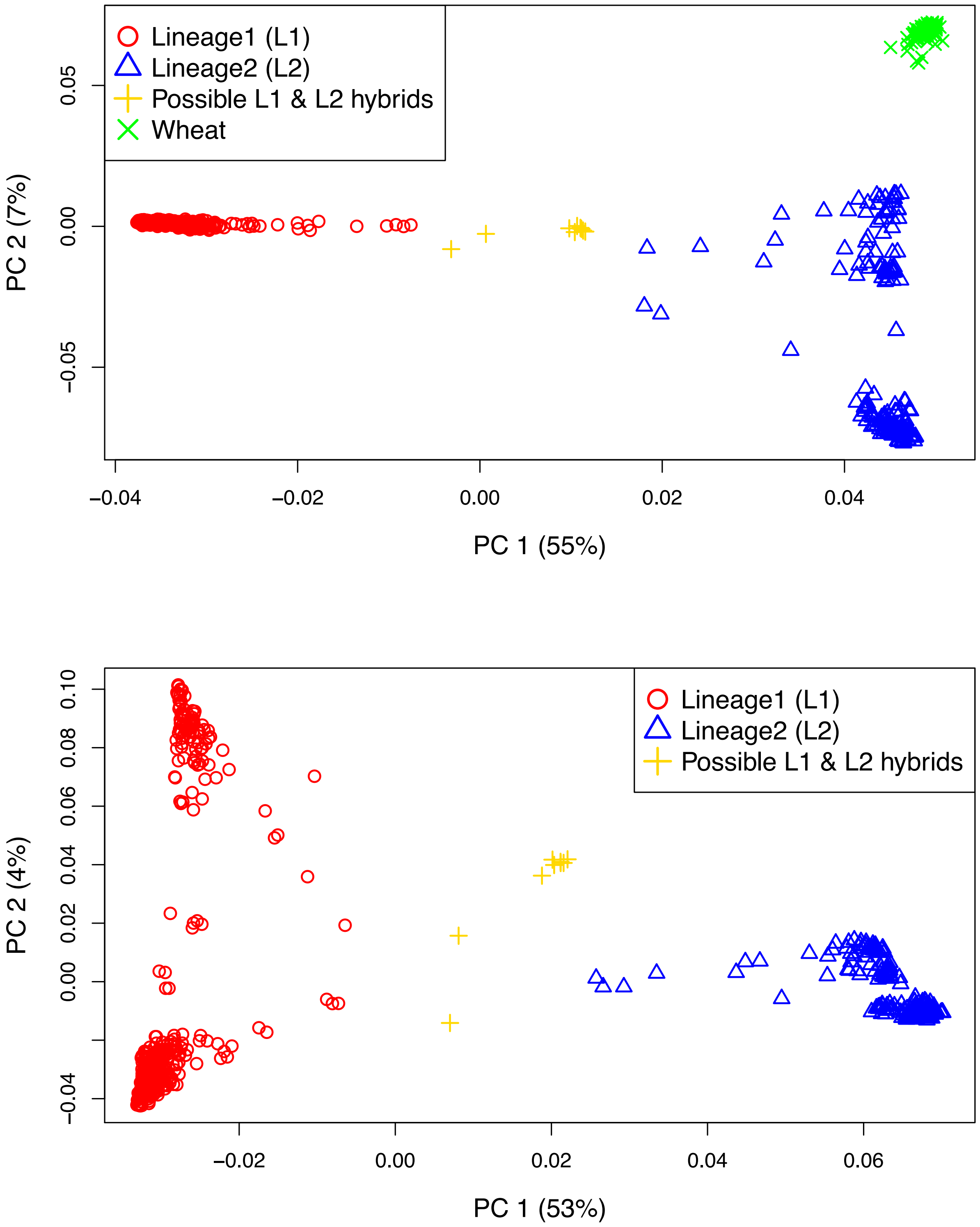

Figure S5. Corrplot showing correlation coefficients among different variables and principal component 2 (left), and principal component 3 (right). Size of the circle represent the strength of correlation. Color scale is shown in the right of the plot. Correlation coefficients are shown inside the circles.

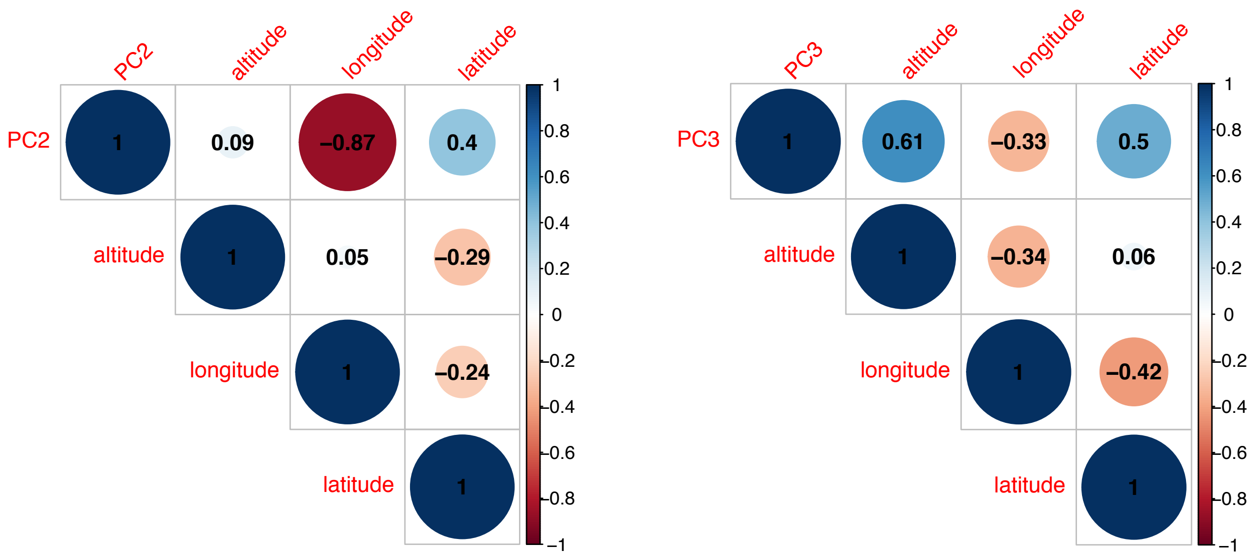

Figure S6. Scatterplot showing the relationship between Altitude of Lineage2 accessions and third principal component. Strong correlation between two variables is evident suggesting that PC3 is separating out lower and higher altitude accessions. Correlation coefficient is shown at the top right corner. Vertical red dotted line marks the 150m altitude that demarcates the lower vs. higher altitude accessions.

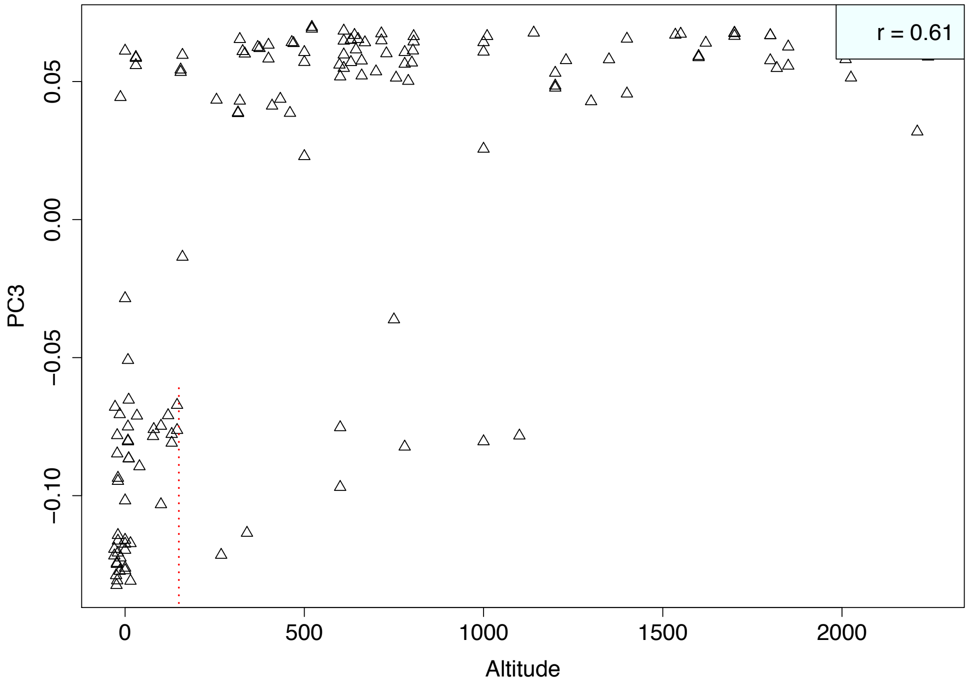

Figure S7. One randomly selected L1 (TA10144) and L2 (TA1664), and putative hybrid accessions' chromosomes showing the distribution of L1 and L2 specific alleles. Red color represents L1 specific alleles, and blue represents L2 specific alleles. Centromeres are marked with thick black bars. Numbers in parentheses in the legend represent the number of lineage specific alleles.

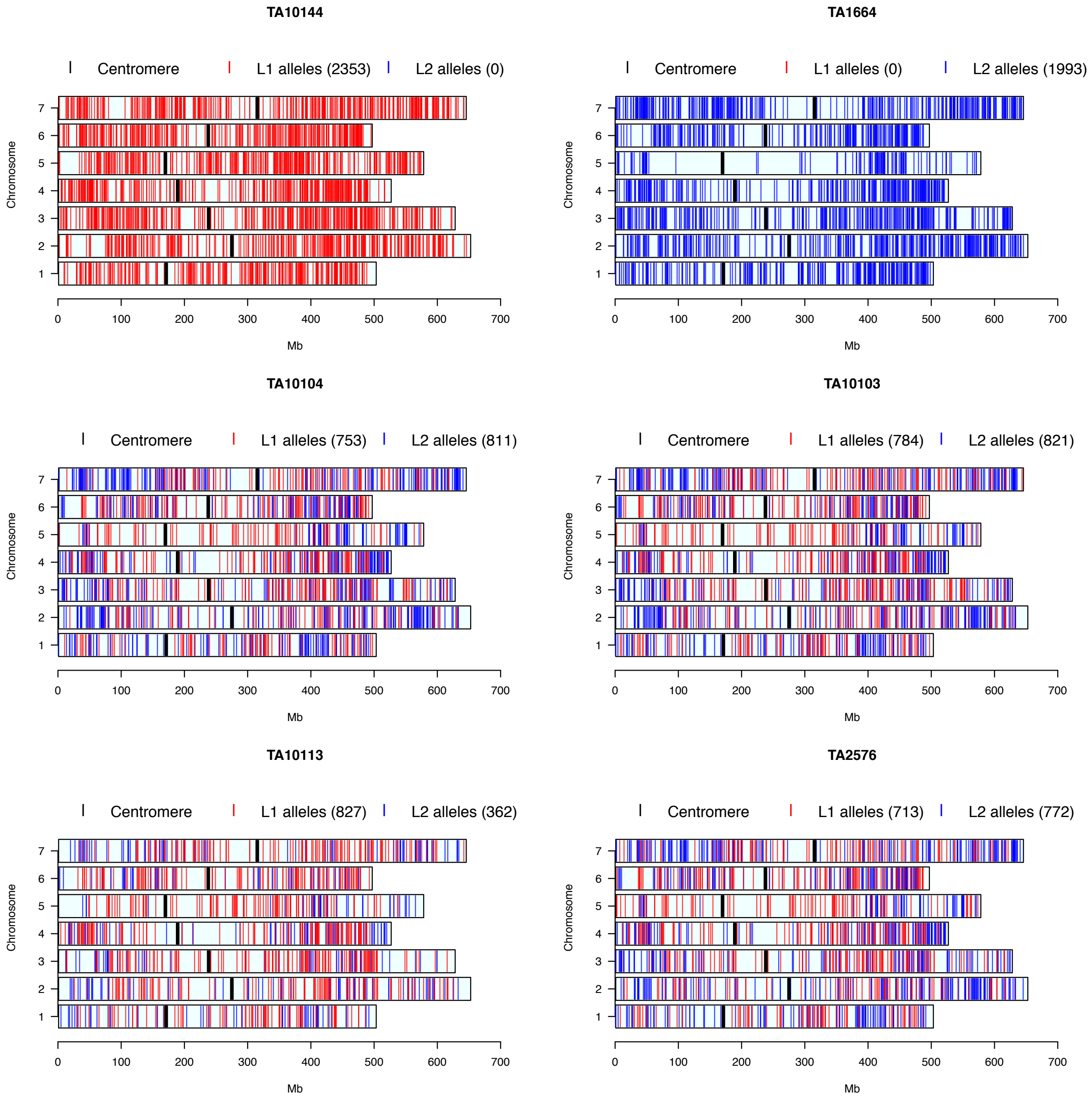

Figure S7. Continued…

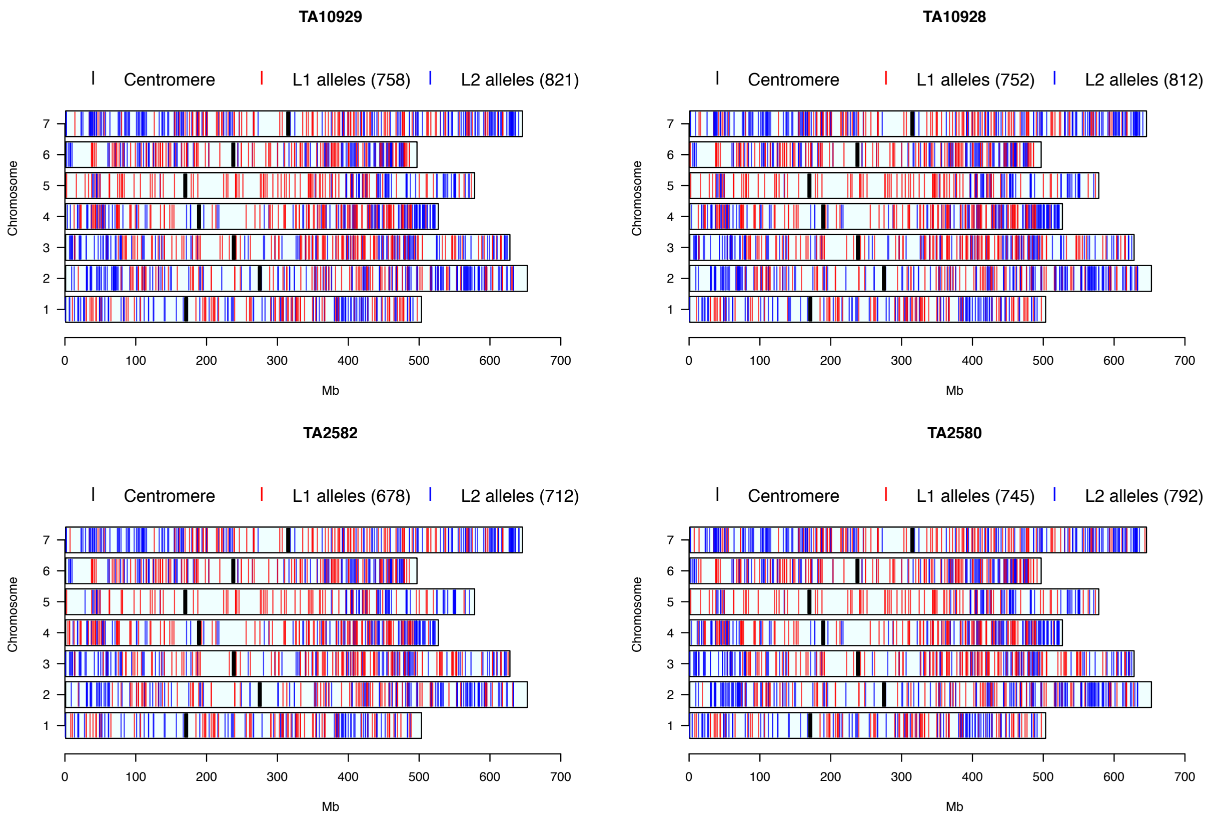

Figure S8. Distribution of lineage specific alleles for 110 segregating SNPs. (Top panel) combined allelic distribution for seven putative hybrids from Georgia and one from Turkmenistan. (Lower panel) allelic distribution for seven putative hybrids from Georgia.

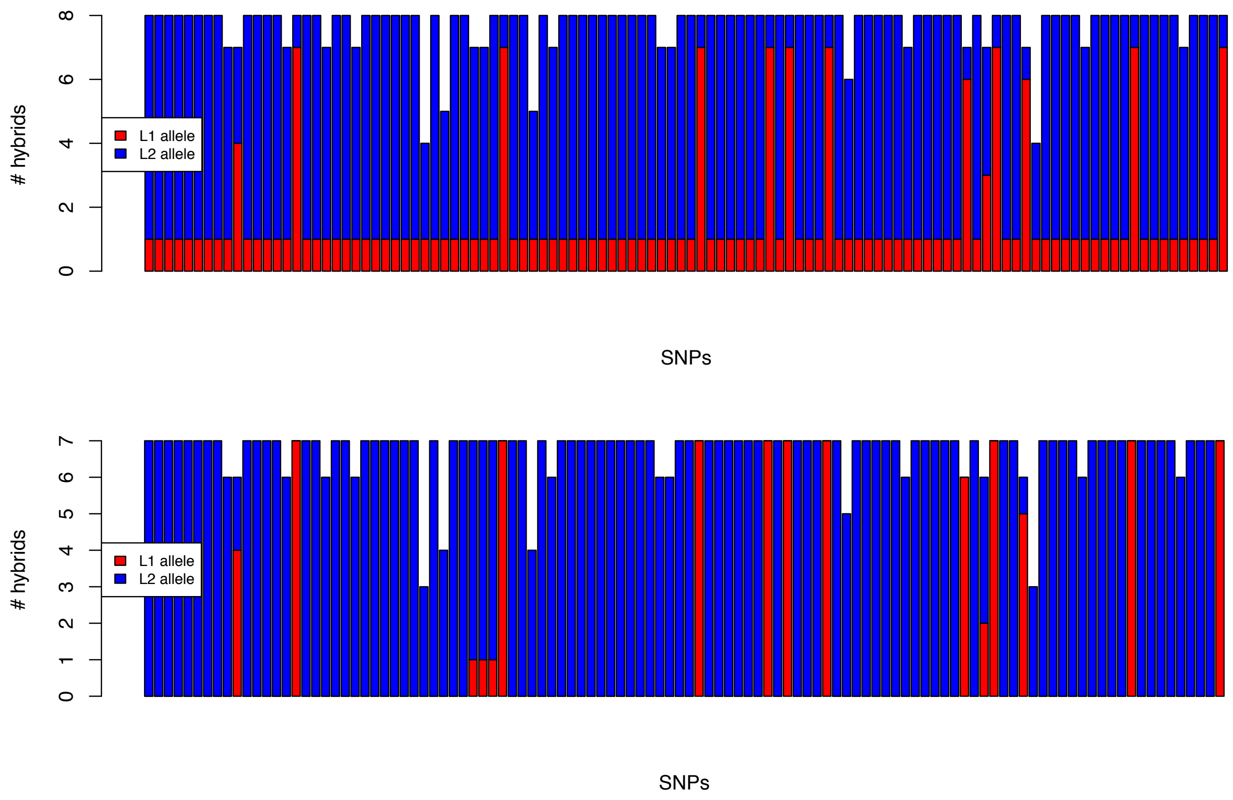

Figure S9. Minor allele frequency histogram for (A) L1 and (B) L2. (C) joint minor allele frequency scatterplot.

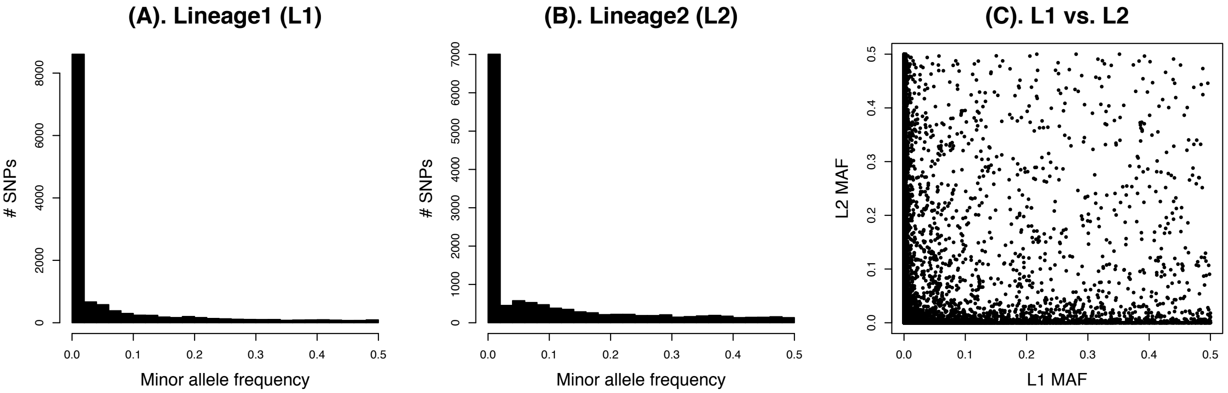

Figure S10. Distribution of minor allele frequencies across all seven chromosomes of *Ae. tauschii*. Red bars represent minor allele frequencies for L1 and blue for L2. Centromere positions are shown as black diamonds.

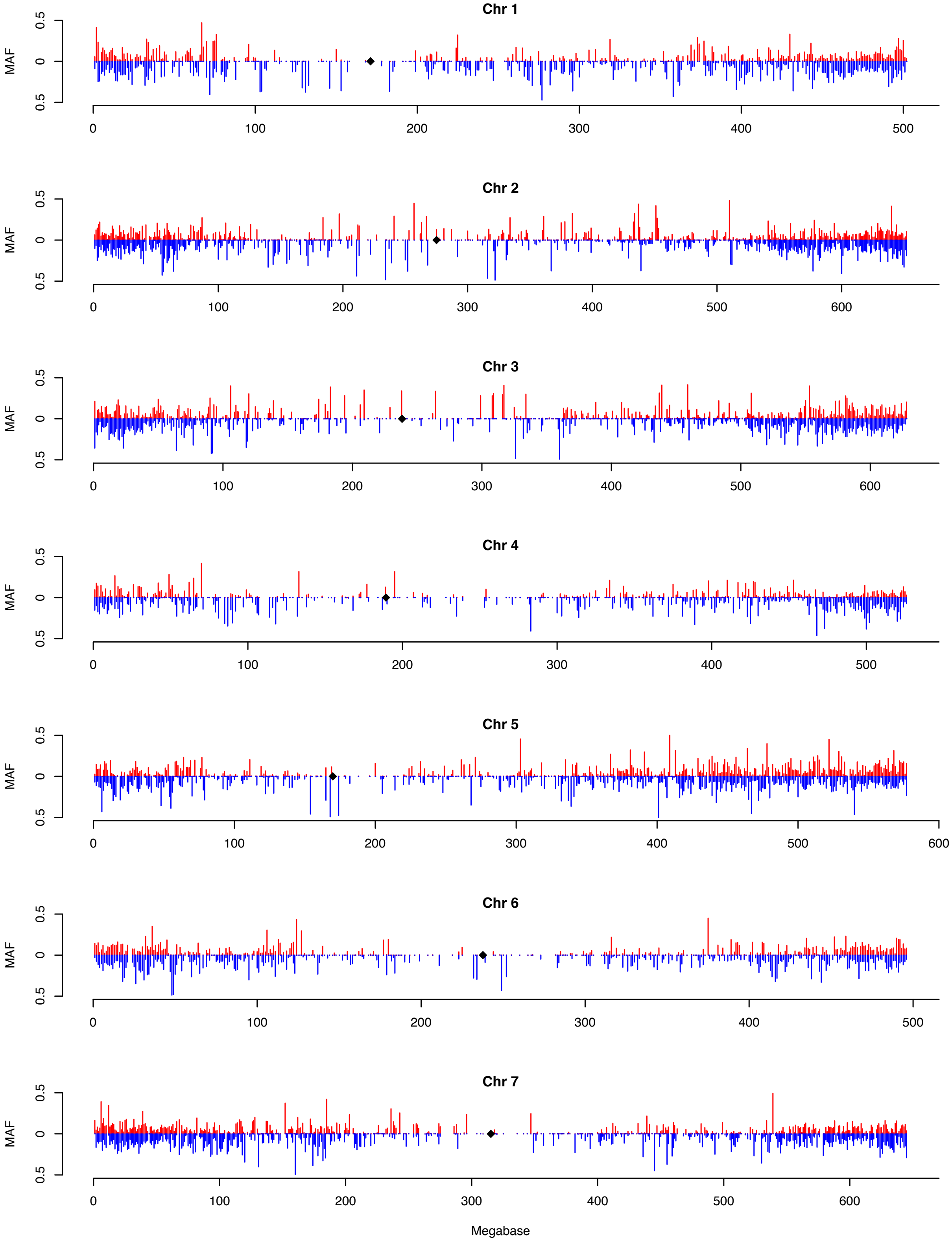

Figure S11. Distribution of MiniCore accessions (red branches) in the whole collection.

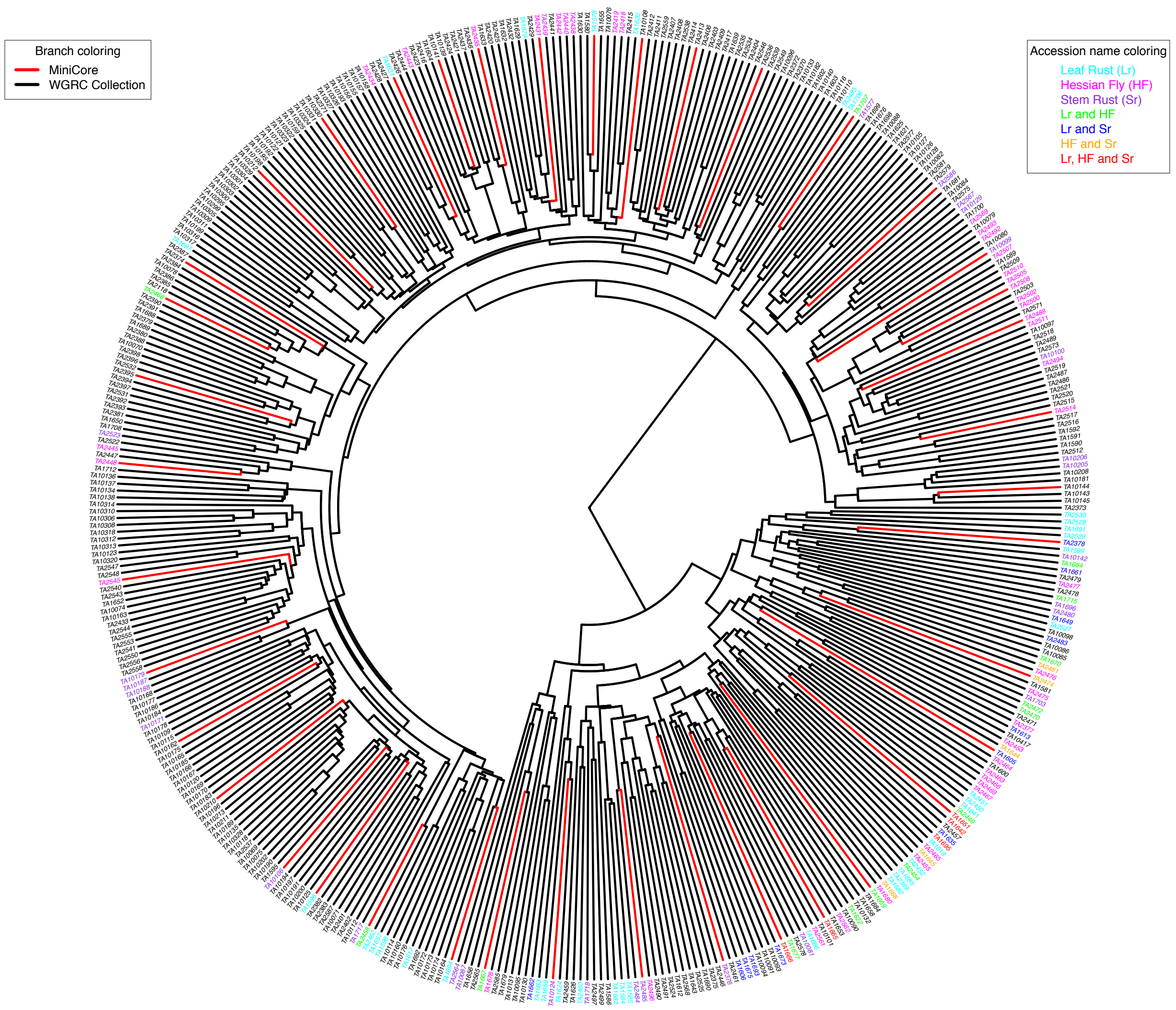

Figure S12. Violin plots showing L1 and L2 distribution for (A) altitude, (B) longitude, and (C) latitude. Red dots are median values.

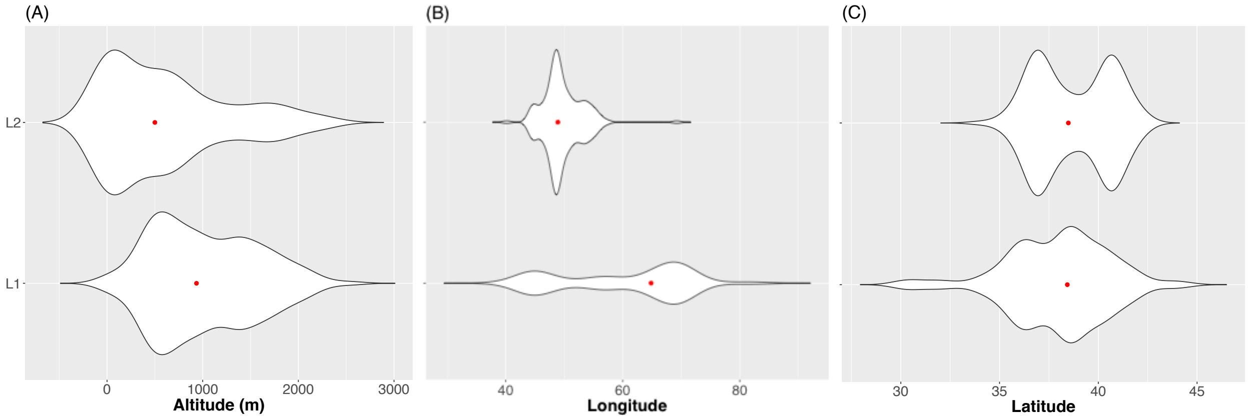

Figure S13. Cytological confirmation of wheat x *Ae. tauschii* F_1 hybrid (n=28; ABDD^t).

A = A-genome of bread wheat, B = B-genome of bread wheat, D = D-genome of bread wheat, D^t = *Ae. tauschii* D-genome. Genomic DNA of *Triticum monococcum*, *Aegilops speltoides*, and *Ae. tauschii* was used as a probe for A, B and D genomes, respectively.

There are seven A-genome (pink), seven B-genome (purple) and 14 D-genome (green) chromosomes. A-genome chromosomes are labeled with FluoroRed Rhodamine, D-genome chromosomes with FITC, and B-genome chromosomes (purple) are counterstained with DAPI.

Full protocol available at WGRC website http://www.k-state.edu/wgrc/images/pdfs/GISH.pdf

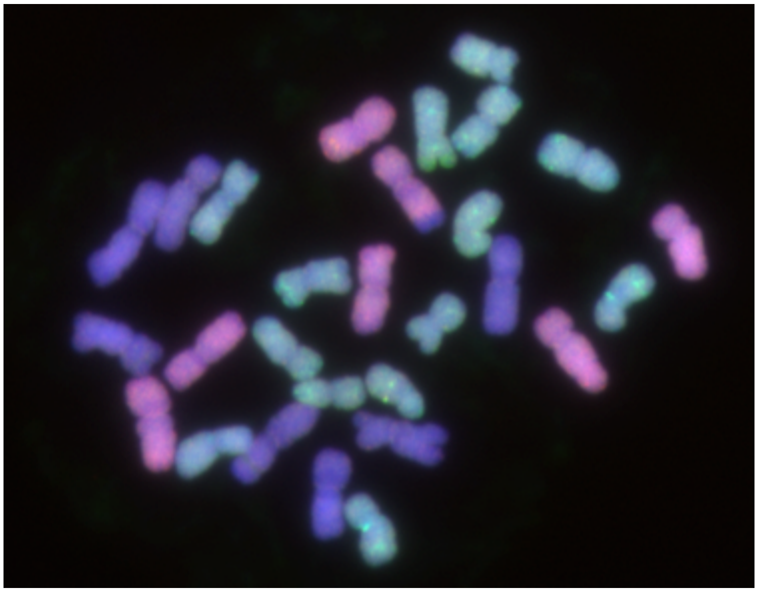

Figure S14. Theoretical change in the genotype frequencies with the advance of generations. Blue lines represent tetrasomic and red lines represent disomic inheritance. Gray vertical dotted lines represent 96% homozygosity threshold for diploid and octo-amphiploid.

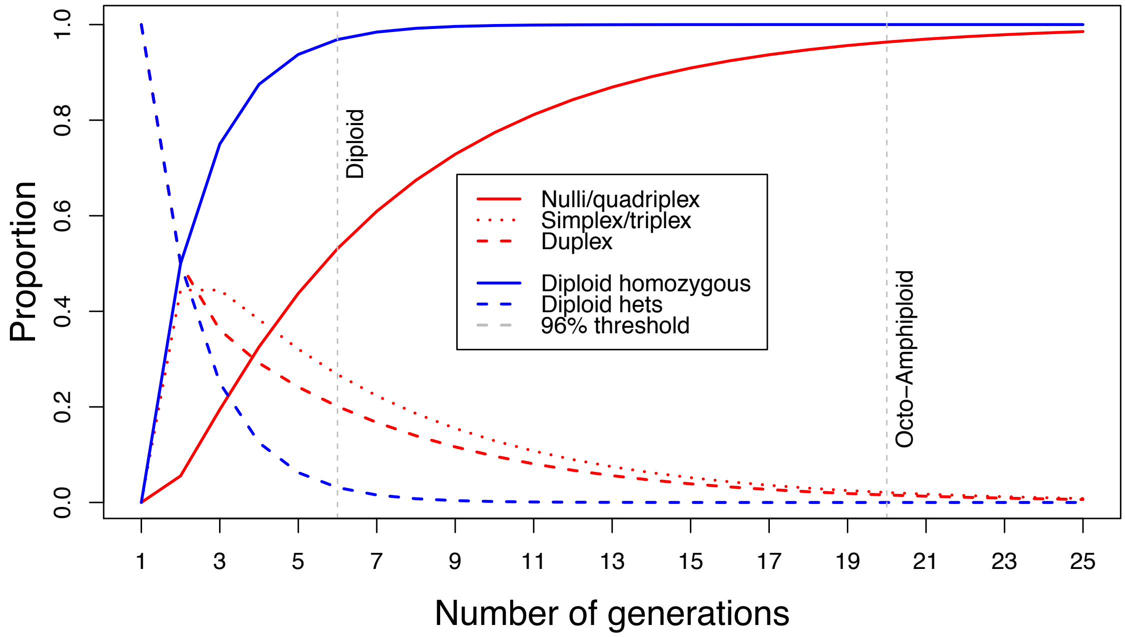

Figure S15. Crossing scheme to retrieve euploid wheat (2n=6x=42). Red arrow represents an *Ae. tauschii* homozygous region in octo-amphiploid.

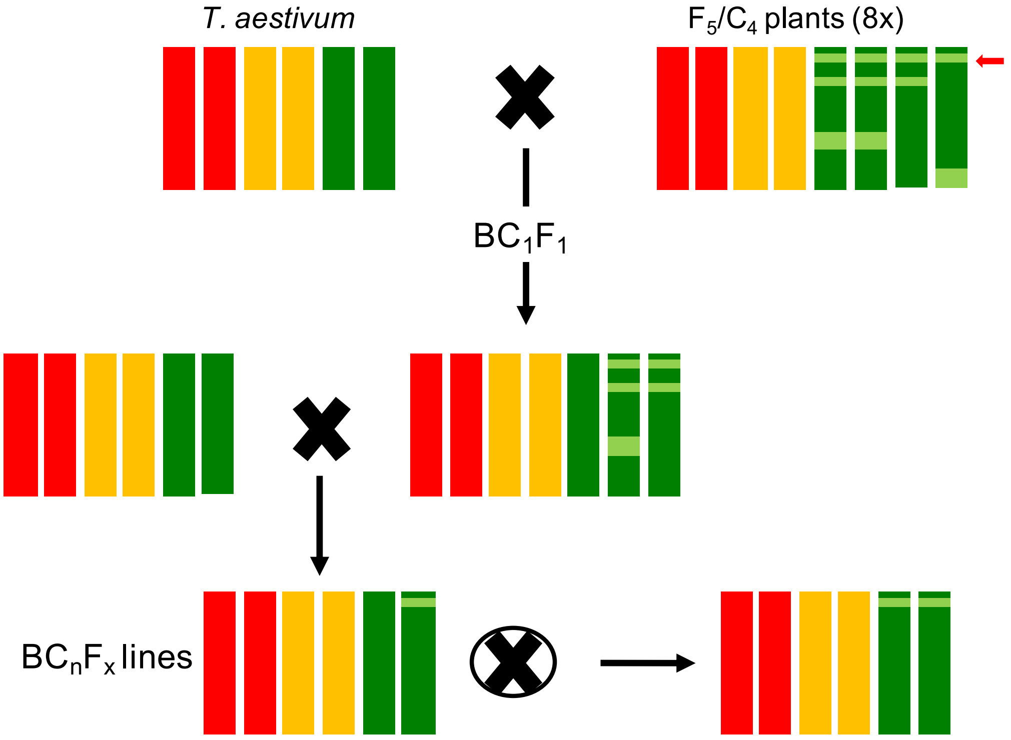

Supplement: Supplementary file 1 [file Data_Sheet_1.PDF]
